# Antioxidant Delivery Revisited: The Promise of Nanostructured Lipid Carriers

**DOI:** 10.3390/medicines13010002

**Published:** 2026-01-22

**Authors:** Leif Behar, Holly Siddique

**Affiliations:** 1 Department of Clinical and Pharmaceutical Science, School of Health, Medicine and Life Sciences, University of Hertfordshire, College Lane, Hatfield AL10 9AB, UK; lb22aaf@herts.ac.uk; 2 Royal Botanic Gardens Kew, Richmond TW9 3AE, UK

**Keywords:** nanostructured lipid carriers (NLCs), antioxidant delivery, bioavailability enhancement, drug delivery systems, natural compounds, controlled release, therapeutic applications, cancer treatment, neurodegenerative diseases, polyphenols

## Abstract

Natural products have an invaluable therapeutic effect on human health. Natural antioxidants, including beta-carotene, turmeric, and polyphenols, are recognised for their health benefits but face significant barriers related to insufficient solubility, instability, volatility, and diminished bioavailability, which limit their therapeutic efficacy in drug delivery systems. Therefore, encapsulation of natural products in a carrier addresses the above concern. Drug delivery systems, such as solid lipid nanoparticles (SLNs) and nanostructured lipid carriers (NLCs), are promising carriers for effective release, consisting of solid and liquid lipids, which enhance efficiency, stability, and controlled release, thereby minimising bioavailability limitations. This review consolidates current studies on the formulation methodologies, mechanisms of action, and therapeutic applications of NLCs, emphasizing their use in the treatment of conditions such as cancer, neurological disorders, and cardiovascular diseases. The results demonstrate that NLCs substantially enhance the bioavailability and therapeutic efficacy of antioxidants, thereby improving their targeted administration and clinical effects. Nonetheless, difficulties in clinical translation remain, including drug loading capacity, regulatory authorisation, and the need for pervasive research on cytotoxicity. This article highlights important areas for future inquiry, specifically the optimisation of NLC formulations, the enhancement of targeting accuracy, and the resolution of safety issues to enhance their clinical application.

## 1. Introduction

Antioxidants play a crucial role in reducing oxidative stress, a condition that contributes to the development of various long-term conditions, including cancer, cardiovascular disease, and neurological disorders. Endogenous and dietary antioxidants are essential for neutralising reactive oxygen species (ROS) and safeguarding cells from oxidative damage. Excessive reactive oxygen species (ROS) production is associated with the development of several degenerative and age-related illnesses, such as cancer, atherosclerosis, neurological disorders, and chronic inflammatory conditions [1]. Antioxidants, including polyphenols, vitamins (such as E and C), and other phytochemicals, may reduce oxidative stress by neutralising free radicals and regulating redox-sensitive signalling pathways. Resveratrol, a natural polyphenolic antioxidant, inhibits NF-κB activation and reduces pro-inflammatory cytokine production, thereby contributing to its protective effects in models of ageing, arthritis, and metabolic conditions [2]. The biological importance of antioxidants is further demonstrated by their proposed use as adjunct therapies to slow disease progression or promote tissue regeneration. Natural antioxidants, including β-carotene, curcumin, and polyphenols, have attracted interest in their potential medicinal benefits.

Nevertheless, despite these beneficial qualities, most antioxidant agents exhibit poor pharmacokinetic properties that limit their therapeutic effectiveness. A prevalent problem is insufficient water solubility, which limits intestinal absorption and reduces oral bioavailability [2]. Numerous antioxidants exhibit chemical instability or are susceptible to degradation; they may be rapidly metabolised and eliminated from the body before attaining therapeutic concentrations in target tissues. Resveratrol highlights these difficulties: it demonstrates significant ROS-reducing and anti-inflammatory effects in vitro; however, it also possesses low long-term stability and is rapidly metabolised, resulting in minimal bioavailability in vivo [2]. Furthermore, naturally occurring flavonoids such as curcumin (a well-known antioxidant derived from turmeric, apigenin, quercetin, resveratrol, and rutin exhibit low water solubility and undergo extensive metabolism, resulting in negligible systemic concentrations following oral administration [3].

**Figure 1:**
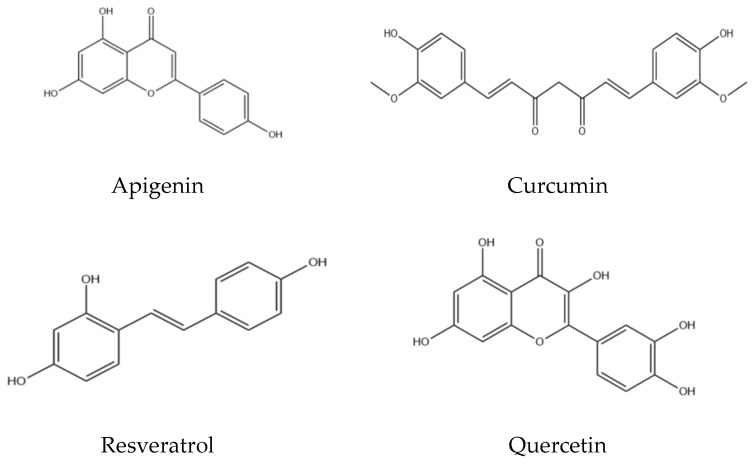
Chemical structures of naturally occurring antioxidants.

These limitations indicate that, despite administering higher doses of antioxidants (Figure 1), only a minimal proportion reaches the circulation or the target site, resulting in subtherapeutic effects. Moreover, certain antioxidants have short half-lives or are sensitive to pH and light, thereby complicating their formulation into standard dosage forms.

To help overcome these constraints, nanotechnology-based delivery methods have been investigated, with Nanostructured Lipid Carriers (NLCs) being an appropriate strategy. NLCs are colloidal particles made up of a combination of solid and liquid lipids, providing benefits over conventional lipid-based systems such as Solid Lipid Nanoparticles (SLNs). The distinct structure of NLCs facilitates increased drug loading capacity, improved stability, and controlled release profiles, making them appropriate for the administration of lipophilic drugs [4]. Their use in administering antioxidants aims to optimise the pharmacokinetic profiles of these substances, thereby improving their medicinal efficacy [5]. The therapeutic efficacy of natural antioxidants is well established; however, their clinical use is frequently constrained by challenges in transport and absorption. NLCs have been studied as carriers capable of overcoming such barriers, enabling efficient delivery of antioxidants to specific sites [4].

The rationale for this study is driven by the increasing interest in employing NLCs to optimise the delivery of antioxidants, therefore improving their therapeutic efficiency.

The rising interest in employing NLCs to improve antioxidant delivery and therapeutic efficacy led to this study. NLCs may deliver antioxidants, although more research is needed. The paper addresses this gap by reviewing the physicochemical properties of NLCs that make them effective drug delivery systems, the formulation strategies used to encapsulate antioxidants in NLCs, the in vitro and in vivo performance of NLC-based antioxidant delivery systems, and the therapeutic applications of NLCs in oxidative-stress-related diseases. These characteristics highlight NLCs’ antioxidant chemical carrier capability and enable their therapeutic usage in oxidative stress-related illnesses.

## 2. Aims of Study

This study seeks to deliver a thorough evaluation of the function of NLCs in improving the bioavailability and therapeutic effectiveness of antioxidants chemicals.

- Through an overview of current studies on the development, characterisation, and use of NLCs in antioxidant administration, this research aims to clarify the ways in which NLCs enhance the pharmacokinetic characteristics of antioxidants.
- Additionally, it aims to clarify the therapeutic potential of NLC-based antioxidant delivery systems in addressing disorders associated with oxidative stress [6].

## 3. Literature Review

### 3.1. Composition and General Structure of NLCs

**Figure 2:**
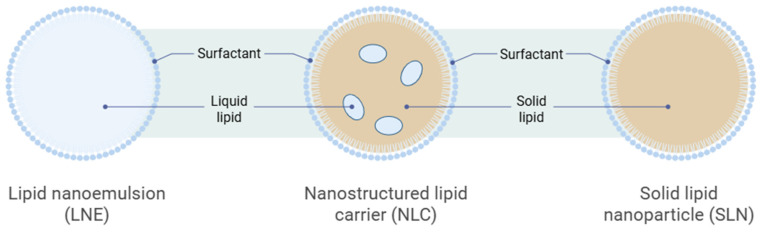
The schematic diagram of the formation of Nanostructured Lipid Carriers (Lipid nano emulsion contains liquid lipid, NLC contains liquid and solid lipid, SLN contains solid lipid only). Image illustrated by BioRender template (https://biorender.com/, accessed on 4 September 2025).

Nanostructured Lipid Carriers (NLCs) are lipid-based nanoparticles designed to enhance medication stability, loading capacity, and controlled release. NLCs consist of an inner core combining solid and liquid lipids, stabilized by emulsifiers at the aqueous interface [7]. This differs from first-generation solid lipid nanoparticles, which consist solely of solid lipids, as shown in Figure 2. The inclusion of liquid lipids (oil) creates a nanostructured matrix with structural imperfections, thereby allowing for a greater bioactive-molecule payload and reducing cargo expulsion during storage [8]. NLCs are typically formulated with biocompatible lipids (e.g., triglycerides, waxes) that remain solid at room and body temperatures, combined with oils (e.g., medium-chain triglycerides, oleic acid), and they form spherical p articles (50–300 nm in diameter) [8]. Surfactants (e.g., Tween 80, poloxamers, lecithin) are added at 0.5–5% *w*/*v* to stabilize the dispersion, reduce interfacial tension, and prevent aggregation [9]. The selection of lipid components and surfactants influences particle size, surface charge, drug-loading efficiency, and colloidal stability.

### 3.2. Internal Core Structure

**Figure 3:**
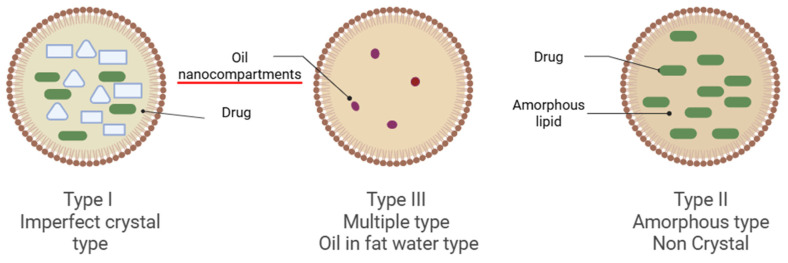
Types of Nanostructured Lipid Carriers are illustrated in the figure. Image illustrated by BioRender template (https://biorender.com/, accessed on 4 September 2025).

Three primary NLC core structures are illustrated in Figure 3. Type I, the Imperfect Crystal Type, involves a small amount of liquid lipid mixed with solid lipid, creating flaws (voids) in the crystalline lattice that can accommodate drug molecules. Type II, the Amorphous Type, is composed of a mix of solid and liquid lipids that remain non-crystalline, preventing ordered lattice formation and minimizing drug expulsion due to lipid recrystallization [7]. Type III, the Multiple Type (O/F/W), contains a larger proportion of oil, allowing nano-sized oil droplets to be trapped in the solid lipid matrix (oil-in-fat-in-water), which solubilizes additional medication, enhances prolonged drug release and reduces leakage.

While all three forms share a hybrid lipid core, they differ in their internal structures, which affect drug loading and release kinetics. Type I imperfect crystals tend to undergo structural breakdown due to solid lipid crystallisation; therefore, they are unstable [10]. On the other hand, in the type II amorphous type, solid lipid constituents subsist in an amorphous state, therefore, exhibit in a disorganised, random configuration [11]. The drug-loading capacity is typically lower than that of imperfect type NLC due to its disordered spatial arrangement [12]. In type III, multiple types of liquid oil nanocompartments provide a drug reservoir. The drug solubility is higher, resulting in enhanced drug loading. The drugs are reserved in an oil phase, thus the drug releases at a slower rate. Accordingly, type III is ideal for controlled drug release administration [13]. A variety of formulation processes, often derived from SLN manufacturing methods, are used to produce NLCs.

### 3.3. Comparison of Nanoparticle Drug-Delivery Platforms with Other Drug Delivery Methods

The study of nanoparticle drug delivery systems encompasses diverse platforms, each characterized by unique structural attributes and performance metrics. NLCs have emerged as a versatile alternative, and a comparison with solid lipid nanoparticles (SLNs), liposomes, and polymeric nanoparticles underscores both the advantages of NLCs and the factors to consider. NLC and SLN have similar physicochemical properties, including lipids and surfactants, making them safe and biocompatible carriers [8]. However, a study [9] highlighted that SLNs can crystallise into ordered structures during cooling and storage, potentially expelling the drug, reducing effective loading, and causing drug precipitation. Additionally, SLNs may form a gel structure at specific concentrations or temperatures, complicating modification [9]. In contrast, NLCs incorporate liquid lipids into the solid matrix, creating a “disturbed” crystal structure that prevents tightly packed configurations, significantly reducing drug expulsion [9]. Studies show that NLCs achieve higher encapsulation efficiencies and maintain drug distribution more effectively during storage than SLNs [8,9].

They were among the first nanoparticle drug carriers and are widely used for both hydrophilic and lipophilic drugs. Liposomes offer enhanced biocompatibility and protect drugs from enzymatic degradation [8]. However, the research [9] highlighted challenges, including relatively low drug loading for lipophilic molecules (which integrate into the bilayer in limited quantities), susceptibility to leakage, and short circulation half-lives due to recognition and elimination by the reticuloendothelial system.

Polymeric nanoparticles (PNPs), including those made from PLGA (poly lactic-coglycolic acid)) or other biodegradable polymers, have been widely researched for drug delivery, including antioxidants. PNPs provide precise control of drug release via polymer degradation and offer excellent stability. However, polymer-based systems often require organic solvents for formulation (e.g., emulsification-solvent evaporation techniques), and residual solvents or by-products may pose toxicity risks. In contrast, NLCs are synthesised from lipids considered generally recognized as safe (GRAS) and typically use surfactants and water in production, avoiding harmful solvents [8,9]. This safety advantage makes NLCs less likely to elicit cytotoxic or inflammatory reactions than certain polymeric carriers.

### 3.4. Antioxidant Drug Delivery and Cellular Uptake

Nanostructured lipid carriers (NLCs) are designed to encapsulate antioxidants to regulate their release, thus enhancing targeted delivery. NLC releases a drug in a biphasic manner, which consists of both an initial rapid release (burst) of surface-localised or loosely bound antioxidants, followed by a slower, sustained release as the antioxidant diffuses outward or the lipid degrades [14]. NLCs can be optimised to modify release kinetics by adjusting the solid-to-liquid lipid ratio. For example, Aditya and his colleagues conducted an in vitro study comparing NLC- and SLN-loaded quercetin drug delivery systems. The studies highlighted superior NLC drug-release performance after 2 h. The results show similar quercetin bioaccessibility after 30 min, at 31% for SLN and 36.7% for NLC. Still, NLC demonstrated significantly higher quercetin bioaccessibility (52.7%) than SLN-based quercetin drug delivery (39.7%). SLNs decrease the hydrolysis rate and intestinal absorption capacity due to their dense, tight structure, although they support controlled lipid digestion. NLC’s disorganised lipid matrix demonstrated a superior result, achieving 79% compared with SLN at 53% overall [15]. Administration of NLCs via the oral route is subject to lipases and various enzymes capable of lipid digestion. Oral administration of antioxidants encapsulated in NLCs is advantageous because intestinal lipases gradually degrade the lipid matrix of NLCs, potentially releasing the antioxidants in the gastrointestinal tract, thereby facilitating absorption. Digested lipids from NLCs may enter normal absorption pathways and indirectly contribute to chylomicron formation, lymphatic transport, a key delivery system for highly lipophilic substances. Instead of being quickly transported to the portal blood and liver for first-pass metabolism, antioxidants in NLCs are absorbed into chylomicrons within enterocytes and enter the lymphatic circulation [16,17]. This lymphatic pathway significantly enhances the bioavailability of antioxidants by bypassing hepatic first-pass metabolism, thereby allowing them to enter the systemic circulation via the thoracic duct.

At the cellular level, NLCs enhance antioxidant delivery by improving cellular uptake. Nano-sized carriers are efficiently internalised by cells through endocytic mechanisms. Encapsulation in NLCs protects antioxidants from early degradation in the bloodstream or extracellular environment; for example, an NLC can shield a polyphenol from oxidation or conjugation with glucuronides/sulfates until it reaches target cells. Once inside cells, the slightly acidic environment of endosomes or the presence of lipases can facilitate the release of antioxidants from NLCs, thereby making them bioavailable at the intended site. This targeted delivery enhances therapeutic efficacy. A notable example is NLCs engineered for cerebral delivery: studies show that modifying NLC surfaces (e.g., with transferring or other targeting ligands) enables receptor-mediated transcytosis across the blood-brain barrier, allowing antioxidants to reach the brain that would not usually penetrate [18].

### 3.5. Therapeutic Applications of Antioxidant-Loaded NLCs

Antioxidant-loaded NLCs are being investigated in cancer, cardiology, neurology, and dermatology because of their capacity to enhance antioxidant stability, bioavailability, and tissue targeting, consequently augmenting therapeutic efficacy in each field.

In oncology, antioxidant-encapsulated nanostructured lipid carriers (NLCs) are gaining recognition as complements to conventional treatments. Tumor cells have abnormal redox balance, while many cytotoxic treatments work by producing reactive oxygen species (ROS). Exogenous antioxidants can sensitise cancer cells while protecting healthy tissue from ROS damage [19]. However, polyphenols such as curcumin and quercetin have low solubility, rapid metabolism, and short circulatory half-lives, which limit their efficacy. Encapsulation in NLCs protects the antioxidant, improves dissolution, extends blood circulation, and targets tumors through the enhanced penetration and retention effect, boosting antitumor efficacy and reducing systemic toxicity in mice [20].

Oxidative stress and inflammation are significant contributors to cardiovascular diseases, including atherosclerosis, hypertension, and myocardial ischemia. Excessive reactive oxygen species (ROS) disrupt endothelial signalling, oxidise lipids, and damage myocardial tissue; thus, enhancing antioxidant defenses represents a rational cardioprotective strategy [21]. Nutraceuticals such as resveratrol, coenzyme Q10, and carotenoids exhibit poor solubility, rapid clearance, and less than 1% oral bioavailability, which restricts their effectiveness [21]. Encapsulating these agents in nanostructured lipid carriers (NLCs) protects them from enzymatic degradation and prolongs their circulation half-life. Studies have shown that resveratrol-loaded NLCs exhibit good stability, with a zeta potential of −25.6 mV, attributable to electrostatic repulsion [21]. Neurodegenerative disorders such as Alzheimer’s disease (AD) and Parkinson’s disease (PD) are intricately associated with oxidative stress and neuronal injury. The brain’s elevated oxygen consumption and lipid composition render it particularly susceptible to oxidative damage, characterized by increased reactive oxygen species and compromised antioxidant defenses in Alzheimer’s disease, Parkinson’s disease, and associated illnesses [22]. Antioxidants such as curcumin, quercetin, and certain polyphenols have demonstrated neuroprotective effects by scavenging free radicals, chelating metal ions, and modulating cell-survival pathways. However, delivering antioxidants to the central nervous system (CNS) is challenging due to the blood-brain barrier (BBB), which limits the uptake of many compounds. Nanostructured lipid carriers (NLCs) overcome this by facilitating BBB penetration or enabling alternative routes, such as intranasal delivery, while their nanoscale size enables neuronal and glial cell uptake [23]. NLCs allow prolonged release in brain tissue, preserving therapeutic levels of antioxidants that would otherwise be rapidly eliminated [24]. An example is curcumin-encapsulated nanostructured lipid carriers for the treatment of Alzheimer’s disease. Curcumin, a powerful antioxidant and anti-inflammatory polyphenol, impedes amyloid-beta aggregation and diminishes oxidative damage, although it has limited brain bioavailability in its free form. Malvajerd and his colleages (2019) [24] encapsulated curcumin in nanostructured lipid carriers to enhance its transport to the brain in an Alzheimer’s disease rat model. Their formulation markedly enhanced curcumin accumulation in the rat brain (exceeding 500 ng/g after 1 h, substantially surpassing free curcumin) and boosted serum concentrations, demonstrating effective systemic absorption and blood-brain barrier transit. Rats administered curcumin NLCs exhibited diminished oxidative stress markers in the hippocampus (reduced ROS production, lipid peroxidation, and normalised ADP/ATP ratio) and improved cognitive function in maze-based assessments compared with those receiving unencapsulated curcumin. This in vivo study indicated that NLCs can surmount delivery obstacles in neurodegeneration, enabling antioxidants to attain therapeutic concentrations in the brain and alleviate symptoms. Likewise, silibinin-loaded nanostructured lipid carriers in Alzheimer’s disease models demonstrated enhanced memory retention and neuroprotective benefits relative to free silibinin, attributed to superior brain absorption [25].

Subsequent investigations, encompassing clinical trials, will determine whether these results can apply to humans. The existing research robustly supports the therapeutic efficacy of NLC-mediated antioxidant administration in slowing neurodegeneration and improving neurological function.

### 3.6. Limitations and Safety Considerations of NLCs

Nanostructured lipid carriers (NLCs) enhance antioxidant delivery; however, their efficacy is limited by several drawbacks. Although drug loading exceeds that of solid-lipid nanoparticles, it reaches a limit beyond which excess drug may crystallize or escape [9,26]. The release often follows a biphasic pattern, with an initial surge followed by gradual dispersion, which can hinder the attainment of controlled-release goals [27]. Storage stability remains an issue because particles may agglomerate and lipids can undergo polymorphic transitions, thereby reducing entrapment efficiency and distorting release profiles. While incorporating liquid lipids reduces crystallinity and ejection, it doesn’t fully prevent phase separation or recrystallisation. Long-term stability depends on carefully calibrated lipid ratios and sufficient surfactant [28].

Safety considerations are paramount in NLC development. Most NLC lipids and surfactants are generally recognised as safe (GRAS); however, nanoscale processing may raise safety concerns. At high concentrations, colloidal-stability surfactants can induce tissue irritation or cytotoxicity, and even safe lipids such as medium-chain triglycerides can produce hazardous metabolites after digestion [29]. Thus, before clinical usage, each new NLC formulation must undergo in vitro and in vivo toxicity assessment. Antioxidant-loaded NLCs had low acute toxicity and immunogenicity, according to studies. They can pass the intestinal epithelium and the blood-brain barrier due to their tiny size. Unmodified NLCs are rapidly opsonised and sequestered by the mononuclear phagocyte system, leading to accumulation in the liver and spleen and potentially impairing therapeutic efficacy and raising concerns about long-term deposition. PEGylation, a “stealth” coating, which is often used for improving NLCs’ drug and gene delivery [30] may delay clearance yet provoke immunological responses [29].

Eventually, NLCs face significant production and regulatory challenges. Minor homogenization or temperature changes may cause batch-to-batch fluctuations in particle size and drug content, making scale-up from laboratory to industrial scale challenges. Consistency requires quality-by-design and cGMP process control, with real-time monitoring. Batch reproducibility using high-pressure homogenisation and microfluidics [31]. NLCs require comprehensive characterisation, toxicity testing, and regulated manufacture before approval. As a result, only a few NLC-based cosmetics have been released [9]. Regulatory clearance requires standardisation of nanocarrier assessment and mitigation of chronic exposure [29]. For successful antioxidant therapy, researchers must improve NLC stability, safety, and scalability.

## 4. Result

### 4.1. Characterisation of NLCs

#### 4.1.1. Key Physicochemical Parameters and Analytical Methods

A comprehensive physicochemical characterisation of NLCs is crucial to guarantee their quality, efficacy, and consistency, particularly for therapeutic uses. Essential parameters involve particle size and distribution, surface charge, morphology, drug encapsulation efficiency, and the physical state of the lipid matrix. A range of analytical methods is utilised to characterise these attributes of NLC formulations [8].

#### 4.1.2. Particle Size and Size Distribution

The size of NLCs (typically between 50–300 nm) is commonly assessed using dynamic light scattering (DLS), which provides the intensity-weighted hydrodynamic diameter and the polydispersity index (PDI) of the nanoparticle ensemble. A smaller size is preferred to increase surface area and enhance cellular uptake, whereas a low PDI (≤0.3) indicates a homogeneous size distribution and reduced aggregation. Nanoparticle tracking analysis (NTA) allows direct visualisation and sizing of individual particles in suspension, often complementing DLS [14]. Particle size regulation is crucial because it affects drug release (smaller particles have a greater surface area, leading to faster release) and biodistribution (particles under ~5 nm may avoid blood vessels or be eliminated renally, whereas those over 200 nm may be more likely to be phagocytosed by macrophages). Studies show that NLC sizes typically approximate 100 nm with narrow distributions, achieved through optimized homogenization and surfactant concentrations [14].

#### 4.1.3. Surface Charge (Zeta Potential)

The zeta potential (surface charge) quantifies the surface charge of NLCs in suspension, typically measured by electrophoretic light scattering. NLCs often exhibit a negative zeta potential (e.g., −10 to −40 mV) due to ionized fatty acids or anionic surfactants on their surface [8]. For example, an NLC formulation may exhibit a zeta potential of approximately −12 mV when stabilised with non-ionic surfactants, or a more negative value (e.g., −30 mV) with ionic stabilisers such as sodium glycocholate. Zeta potential indicates colloidal stability; particles with a high zeta potential (typically exceeding |30| mV) resist aggregation due to electrostatic repulsion [8]. Neutral or low-charge NLCs may require steric stabilizers (e.g., Poloxamer or PEGylation) for stability. Characterizing zeta potential provides insights into the formulation composition and the success of surface modifications, such as coating with a cationic polymer or targeting ligand. A slightly negative zeta potential can be beneficial for specific biological interactions, whereas cationic NLCs (positive zeta potential) may enhance cellular uptake but also increase cytotoxicity.

### 4.2. Morphology and Surface Characteristics

The morphology and surface characteristics of NLCs are typically analysed using electron microscopy. Transmission electron microscopy (TEM), often with cryo-TEM to observe particles in a vitrified state, provides high-resolution images that confirm the round shape and smooth surface of NLCs [32]. TEM can sometimes reveal the core–shell structure if there is contrast between the core and surfactant corona, though this distinction may be less pronounced in NLCs compared to metal-core nanoparticles. Scanning electron microscopy (SEM) can visualize particle surface topology, but TEM is more commonly used for nanoscale lipid carriers. Atomic force microscopy (AFM) is another technique that can image nanolayered composites and measure surface roughness. Microscopy images confirm the successful formulation of discrete nanoscale particles and can identify any larger anomalous structures or coalescence. TEM micrographs of well-prepared NLCs typically show spherical particles with diameters matching DLS measurements, with no visible drug crystals outside the particles [32].

### 4.3. Encapsulation Efficiency and Drug Loading

Encapsulation efficiency measures the proportion of the initial antioxidant drug effectively encapsulated within the NLC compared to the unencapsulated or lost amount. This is determined by isolating the NLCs from the unbound drug via ultracentrifugation, filtration, or dialysis, and then quantifying the drug content using HPLC or UV spectrophotometry. High encapsulation efficiency (typically over 80% for well-optimized nano lipid carriers) ensures that most of the antioxidant is transported by the nanoparticles [8]. Drug loading, defined as the amount of drug relative to the total nanoparticle weight, is a key metric typically expressed as a percentage. Efficient NLC formulations for antioxidants exhibit encapsulation efficiencies of 85–99%, indicating minimal drug loss. An optimized resveratrol NLC showed an encapsulation efficiency of about 98%, due to the high solubility of resveratrol in the lipid blend and the inclusion of liquid lipid to improve accommodation. Low EE may suggest drug incompatibility with the lipid matrix or the need to adjust the formulation process (e.g., cooling rate, surfactant type) to prevent drug crystallisation outside the particles [8].

### 4.4. Lipid Matrix Crystallinity and Polymorphism

Since NLCs consist of both solid and liquid lipids, it’s crucial to characterise the crystallinity and polymorphic state of the lipid matrix. Techniques such as differential scanning calorimetry (DSC) and X-ray diffraction (XRD) assess whether the lipid in NLCs is less crystalline than in the pure solid lipid. An effective NLC formulation typically exhibits a lower melting point and reduced crystallinity (wider, less intense XRD peaks) than the bulk lipid, indicating the presence of liquid lipid and an amorphous matrix [7]. DSC thermograms may reveal a broader thermal event rather than a distinct melting endotherm for the solid lipid, indicating the formation of a nanostructured lattice that enhances drug accommodation. Characterising these properties is important, as excessive lipid recrystallisation can lead to antioxidant expulsion. Stability studies evaluate NLC size, zeta potential, and drug content over time under different storage conditions (e.g., 4 °C, room temperature). A stable NLC exhibits minimal changes in particle size and no significant drug degradation or leakage over months [8].

### 4.5. Stability and In-Vitro Release Studies

One study demonstrated that an NLC maintained its size and preserved over 90% of the drug after three months at room temperature [33]. This data is crucial for converting these systems into physical pharmaceutical products. Furthermore, in vitro release studies, employing techniques such as dialysis bags or Franz diffusion cells, are routinely performed to evaluate the antioxidant release profile from NLCs under sink conditions or biorelevant media [14]. These investigations often demonstrate a slower release of NLCs compared with the free drug, and kinetic modelling (e.g., Korsmeyer-Peppas or Higuchi models) elucidates the release process (diffusion-controlled, erosion-controlled, etc.).

A thorough evaluation of NLCs’ size, charge, morphology, encapsulation, and lipid state offers insight into the formulation’s quality and its anticipated behaviour in biological systems. These characterisations ensure that the antioxidant-loaded NLCs are appropriately designed for their intended use, exhibiting consistent properties that support efficacy and safety.

Previously reported physicochemical parameters of several natural antioxidants are listed below; these parameters indicate smaller particle size, enhanced stability, increased drug-loading capacity, and improved bioavailability upon encapsulation in an NLC carrier.

## 5. Discussion

The findings underscore the considerable potential of nanostructured lipid carriers (NLCs) as sophisticated delivery vehicles for antioxidant chemicals. A persistent issue in the therapeutic application of naturally occurring antioxidants, such as curcumin, resveratrol, Lutein, Astaxanthin, and quercetin, is their inadequate water solubility, diminished oral bioavailability, and vulnerability to degradation under physiological conditions [34]. These constraints hinder their clinical effectiveness and require novel medication delivery methods. The evidence indicates that NLCs mitigate these limitations by providing a nanostructured matrix that encapsulates and stabilises antioxidants, preserves components from degradation, and enhances their bioavailability via improved solubility and lymphatic transport [35,36,37]. Several studies (Table 1) have shown that NLC generally exhibit smaller particle sizes and lower polydispersity indices (PDIs) than solid lipid nanoparticles (SLNs). Similarly, zeaxanthin-loaded NLCs have been reported to have smaller particle sizes and lower PDI values than their SLN counterparts [38]. In another study, epigallocatechin gallate-loaded SLN and NLC exhibited particle sizes of 364  ±  11 nm and 300  ±  7 nm, respectively, while their PDIs were 0.19  ±  0.03 (SLN) and 0.15  ±  0.02 (NLC) [39]. These findings consistently indicate that NLCs exhibit a more homogeneous particle-size distribution. This effect can be attributed to structural differences between the two systems: SLNs often form highly ordered crystalline lipid matrices, resulting in more rigid and dense particles that can contribute to larger particle sizes [40].

SLNs and NLCs improve stability during gastrointestinal transit. In quercetin-loaded systems, particle sizes remained small in gastric fluids but increased in intestinal fluids due to lipase activity. After 120 min, NLCs exhibited higher bioaccessibility than SLNs [15]. Curcumin-loaded SLNs were stable under acidic conditions but degraded by 8–10% in intestinal fluids, yet still provided better protection than free curcumin [49]. For lutein-loaded SLNs and NLCs with zein-peptide stabilisers, particle size increased after gastric digestion due to enzyme activity, pH or ionic strength. The particle size decreased in the small intestine relative to the acid condition, indicating enzymatic degradation of proteins and lipids during digestion [50].

Dissolution and pharmacokinetic studies demonstrate that solid lipid nanoparticles (SLNs) and nanostructured lipid carriers (NLCs) can enhance drug release and bioavailability. Curcumin-loaded SLN exhibited a sustained, zero-order release for up to 120 h with nearly complete release 99% [49], while β-carotene-loaded SLN showed an initial burst within one hour followed by controlled release for 48 h [51]. The release behaviour of quercetin and zeaxanthin was enhanced in NLC due to their flexible lipid matrix, whereas the rigid crystalline structure of SLN often hindered release. In contrast, SLN loaded with epigallocatechin gallate and trans-ferulic acid exhibited higher drug release than NLC, likely due to crystallinity-induced drug expulsion; however, NLC provided a more sustained profile by minimising expulsion through liquid lipid incorporation [15,39]. Pharmacokinetic data further confirmed improved bioavailability: β-carotene-loaded SLN doubled serum levels in rats compared with free β-carotene, and curcumin-loaded SLN in humans achieved detectable systemic exposure, whereas the curcumin extract did not. Similarly, trans-ferulic acid-loaded SLNs and NLCs increased plasma concentrations and accumulated predominantly in the intestine, with NLCs outperforming SLNs. These findings highlight the potential of SLN and NLC as effective carriers for sustained release and enhanced oral bioavailability of bioactive compounds. However, NLCs provide sustained release and improved absorption, particularly in the intestine, due to their liquid lipid content [52].

The comparison with alternative nanocarriers, solid lipid nanoparticles, liposomes, and polymeric nanoparticles, illustrates that nanostructured lipid carriers offer a distinctive equilibrium among biocompatibility, stability, and drug-loading efficacy. Although liposomes are highly efficacious for hydrophilic antioxidants and provide biocompatibility, their restricted physical stability and susceptibility to drug leakage over time diminish their long-term therapeutic dependability [53]. Polymeric nanoparticles, although providing customized release profiles and structural integrity, sometimes require intricate synthesis procedures and the use of potentially hazardous chemical solvents, which may jeopardize safety [19]. NLCs address these issues by utilising physiological lipids and scalable production techniques, while providing sustained antioxidant delivery and improving therapeutic indices.

Therapeutically, NLCs have demonstrated superior outcomes across several disease models. In oncology, antioxidant-encapsulated nanostructured lipid carriers, such as those with curcumin, have enhanced cytotoxicity against malignant cells while reducing harm to healthy tissues [54]. In cardiovascular illness, resveratrol-NLCs restored endothelial function and decreased oxidative stress in hypertensive animals [22]. In Alzheimer’s disease models, curcumin-NLCs crossed the blood-brain barrier and improved cognitive performance [55]. These results emphasise how NLCs improve pharmacokinetics and therapeutic effectiveness. Nonetheless, constraints persist. NLCs pose cytotoxic hazards due to surfactants, immunogenic reactions, and organ accumulation, particularly in the liver and spleen [28,31]. Batch-to-batch variability in production, influenced by factors such as lipid type and homogenization, presents challenges for large-scale manufacturing. Implementing quality-by-design principles and performing long-term stability studies are essential for regulatory approval [32]. Additionally, most supporting evidence derives from preclinical studies. Additional comparative and in vivo research, including human trials, is necessary to confirm the effectiveness and safety of antioxidant-loaded NLCs. Surface-modified or stimuli-responsive nanostructured lipid carriers might enhance targeting and treatment accuracy. Co-delivery techniques that combine antioxidants with chemotherapeutics or anti-inflammatory agents require further investigation.

## 6. Future Directions

Unlike existing reviews that primarily catalogue formulation strategies or report short-term improvements in antioxidant bioavailability using nanostructured lipid carriers (NLCs), this perspective critically evaluates the translational bottlenecks limiting their real-world application. Specifically, it integrates physicochemical instability, long-term safety uncertainty, intranasal nose-to-brain delivery challenges, regulatory fragmentation, and the scarcity of clinical validation into a unified framework. By highlighting underexplored areas such as temperature-induced degradation under extreme climates, clinically relevant animal models for intranasal delivery, synergistic co-encapsulation strategies, and the need for harmonized analytical and regulatory standards, this perspective moves beyond formulation optimization to outline actionable priorities for clinical translation of antioxidant-loaded NLCs.

While nanostructured lipid carriers (NLCs) show strong potential for delivering antioxidant compounds, several critical limitations currently constrain their broader application and define key directions for future research. A primary challenge is the physicochemical stability of antioxidant-loaded NLCs. Many antioxidants—such as polyphenols, carotenoids, curcumin, and flavonoids—are chemically labile and prone to degradation. During storage, NLC formulations may undergo particle aggregation, drug expulsion, and polymorphic lipid transitions, ultimately reducing antioxidant activity and shelf life [12,56]. Although β-carotene, lycopene, resveratrol, curcumin, and quercetin-loaded NLCs demonstrate acceptable physical stability at temperatures between 4 °C and 25 °C for up to three months, significant degradation occurs above 25 °C. This temperature sensitivity underscores the need for formulation strategies that maintain bioavailability and stability under extreme climatic conditions [57,58,59,60].

In addition to stability concerns, safety and biocompatibility issues remain insufficiently understood. The surfactants and excipients used in NLC formulations may pose risks of cytotoxicity, immunogenicity, and long-term tissue accumulation. However, systematic investigations into NLC biodistribution, chronic toxicity, and mechanistic interactions at the cellular and molecular levels are limited, making comprehensive risk assessment difficult [11,61].

Although oral delivery of antioxidant-loaded NLCs has been extensively studied, intranasal delivery of NLCs for central nervous system (CNS) antioxidant therapy remains underexplored. Oxidative stress is a key driver of neurodegenerative diseases, and intranasal administration offers a non-invasive route to bypass the blood–brain barrier (BBB). Emerging evidence suggests that chitosan-coated solid lipid nanoparticles (SLNs) and NLCs significantly enhance nose-to-brain delivery [62,63]. For instance, chitosan-coated BPE-CS-NLCs exhibited a prolonged brain Tmax (Time to reach maximum drug concentration) compared with intranasally administered free drug, indicating sustained release [64]. Similarly, thermosensitive in situ gels (e.g., methylcellulose and poloxamer 407) further enhance delivery, with embedded SLNs demonstrating increased brain Cmax (Maximum (peak) drug concentration) and targeting efficiency [65].

However, most intranasal in vivo studies rely on rat models, which present notable anatomical limitations. In rats, the olfactory region comprises approximately 50% of the nasal cavity, compared with only ~10% in humans, potentially overestimating brain delivery efficiency [66,67]. Moreover, the small anterior nostrils of rats complicate the administration of viscous formulations. Larger animal models—such as rabbits, dogs, sheep, and non-human primates—offer nasal anatomy more comparable to that of humans and should be prioritised in future pharmacokinetic and translational studies [68].

Another emerging opportunity lies in synergistic co-encapsulation strategies. Antioxidants often require complementary mechanisms, such as anti-inflammatory or metal-chelating effects, to achieve meaningful therapeutic outcomes. Co-delivery of antioxidants with anti-inflammatory drugs, neuroprotective agents, or chelators within NLC systems may enhance efficacy through pharmacodynamic synergy. However, rigorous studies demonstrating controlled, independent release profiles and quantifiable synergistic effects remain scarce, warranting deeper investigation.

From a manufacturing standpoint, scalability and reproducibility remain significant obstacles. Maintaining consistent particle size distribution, encapsulation efficiency, and batch-to-batch uniformity during scale-up is challenging. Quality by Design (QbD) approaches have shown promise in identifying critical process parameters and improving reproducibility, and recent studies support their broader adoption in NLC manufacturing [69].

Finally, regulatory and clinical translation barriers persist. Regulatory expectations for NLCs vary across agencies such as the FDA, EMA, and Health Canada, which currently rely on existing pharmaceutical, cosmetic, or food frameworks supplemented with nano-specific characterization requirements [70,71]. The absence of harmonized international standards for defining, characterizing, and evaluating lipid-based nanocarriers results in inconsistent analytical methods and toxicological requirements across jurisdictions [72,73]. Although particle size distribution, zeta potential, entrapment efficiency, polymorphism, and oxidative stability are universally recognized as critical quality attributes, no standardized analytical protocols exist, and results may vary depending on the measurement technique employed (e.g., DLS versus nanoparticle tracking analysis).

Ongoing efforts by ISO Technical Committee 229 and the OECD Working Party on Manufactured Nanomaterials aim to provide more explicit guidance; however, complete global alignment has not yet been achieved, complicating regulatory approval pathways [74]. Moreover, despite extensive preclinical evidence, clinical validation remains limited. Well-designed, adequately powered human trials are urgently needed to confirm the safety, tolerability, and therapeutic benefit of antioxidant-loaded NLCs before they can achieve widespread clinical adoption [17].

## Figures and Tables

**Table 1:** The List of antioxidants encapsulated in the NLC carrier.

Drug Name	Parameter	Result	Preparation Method	Biological Activity	Source
Curcumin	Particle size	146.8 nm	NLC prepared by Hot high-pressure homogenisation, Tween 80, and Soya lecithin used as surfactant and stabiliser.	Brain cancer therapy	[41]
	Polydispersity index	0.18			
	Entrapment efficiency	90.86%			
	Zeta potential	−21.4 mV			
Quercetin	Particle size	129 nm	NLC prepared by Hot high pressure homogenisation, PGFE 10, PGFE 6 & SE 11 used as surfactant.	----	[42]
	Polydispersity index	---			
	Entrapment efficiency	93.5%			
	Zeta potential	−26 mV			
Lutein	Particle size	167 to 390 nm	Nanocarrier synthesised using melting emulsification coupled with high-pressure homogenisation technique, Tween 80 and Soyabean lecithin used as surfactant.	Antioxidant activity	[43]
	Polydispersity index	0.12			
	Entrapment efficiency	88.5%			
	Zeta potential	−29.1 and −34.5 mV			
Beta carotene	Particle size	142.70 nm	NLC prepared by high shear hot homogenisation, Tween 80 used as surfactant	Antioxidant activity	[44]
	Polydispersity index	0.26			
	Entrapment efficiency	91.15%			
	Zeta potential	−24.9 mV			
Lycopene	Particle size	163 nm	NLC prepared by high shear hot homogenisation, 1 g of Eumulgin^@^SG was dissolved in water for aqueous phase	Antioxidant activity cutaneous delivery	[45]
	Polydispersity index	0.14			
	Entrapment efficiency	---			
	Zeta potential	−74.5 mV			
Anthocyanins	Particle size	107 nm	The inner aqueous phase prepared by dissolving 10 mg of the sample in 1.25 mL purified water. The inner aqueous and liquid phases were homogenised.	------	[46]
	Polydispersity index	0.17			
	Entrapment efficiency	60%			
	Zeta potential	−25.6			
Zeaxanthin	Particle size	79–130 nm	Tween 80 emulsifier used for aqueous phase, lipid phase made using lecithin and the sample Zeaxanthin		[38]
	Polydispersity index	0.23–0.30			
	Entrapment efficiency	90%			
	Zeta potential	−22.23 to −16.88 mV			
Astaxanthin	Particle size	85.3–138.3 nm	NLC formulation consists of 5 wt% lipid phase (5 mg lecithin + 20 mg astaxanthin + 975 mg of lipid mixture), 95% aqueous phase	Antioxidant activity	[47]
	Polydispersity index	0.1–0.25			
	Entrapment efficiency	--------			
	Zeta potential	−21.9 to −34.6 mV			
	Particle size	60–57 nm	The sample was dissolved in the oil phase at 75 °C, and Tween 80 was used as the surfactant.		[48]
	Polydispersity index	0.33–0.37			
	Entrapment efficiency	-----			
	Zeta potential	−23.7 mV			

## Data Availability

No new data were created or analyzed in this study. Data sharing is not applicable to this article.
